# Microfluidic Device to Manipulate 3D Human Epithelial Cell-Derived Intestinal Organoids

**DOI:** 10.3390/mi13122082

**Published:** 2022-11-26

**Authors:** Miki Matsumoto, Yuya Morimoto, Toshiro Sato, Shoji Takeuchi

**Affiliations:** 1 Department of Life Sciences, Graduate School of Arts and Sciences, The University of Tokyo, 7-3-1 Hongo, Bunkyo-ku, Tokyo 113-8656, Japan; 2 Department of Mechano-Informatics, Graduate School of Information Science and Technology, The University of Tokyo, 7-3-1 Hongo, Bunkyo-ku, Tokyo 113-8656, Japan; 3 Department of Organoid Medicine, Keio University School of Medicine, The University of Tokyo, 35 Shinano-machi, Shinjyuku-ku, Tokyo 160-0016, Japan; 4 International Research Center for Neurointelligence (WPI-IRCN), The University of Tokyo Institutes for Advanced Study (UTIAS), The University of Tokyo, 7-3-1 Hongo, Bunkyo-ku, Tokyo 113-8656, Japan

**Keywords:** intestinal organoid, enteroid, microfluidics, fusion, perfusion

## Abstract

In this study, we propose a microfluidic organoid-trapping device used to immobilize human intestinal organoids and apply fluidic stimuli to them. The proposed device has a microchannel with a trapping region with wall gaps between the channel walls and the bottom surface, and a constriction to clog the organoids in the channel. Since the introduced culture medium escapes from the gap, organoids can be cultured without excessive deformation by hydrostatic pressure. Owing to the characteristics of the organoid-trapping device, we succeeded in trapping human intestinal organoids in the channel. Furthermore, to demonstrate the applicability of the device for culturing intestinal organoids, we induced organoid fusion to form large organoids by aligning the organoids in the channel and applying fluidic shear stress to the organoids to regulate their surface structures. Therefore, we believe that organoid-trapping devices will be useful for investigating organoids aligned or loaded with fluidic stimulation.

## 1. Introduction

Human organoids are in vitro models used for physiological and developmental research, drug screening, and regenerative medicine [1]. A human intestinal epithelial cell-derived organoid utilized to study microbiota, intestinal secretions, gastrointestinal cancers, and other diseases is a cystic monolayer of polarized epithelial cells [2,3,4]. Human intestinal organoids exhibit two specific features: fusion and surface topography. Interestingly, when several intestinal organoids are in contact, they fuse to form larger lumen structures [5,6,7,8]. Moreover, their lumen, which is the apical side of the organoids, represents the human gut lumen; they have crypts and villus-like structures, leading to individual differences in surface topography [4]. Morphological changes on the surface of the intestine are regulated by chemical gradients and mechanical stress [9,10]. Therefore, to express the two unique features of human intestinal organoids—fusion and surface topography—manipulation techniques to contact organoids and apply stress stimuli to organoids are necessary.

Microfluidic devices have been proposed for culturing cells and tissues under controlled conditions [11,12,13,14]. Organoids can also be manipulated and stimulated using microfluidic devices such as high-throughput drug screening systems and pathological models [15,16]. Manipulation and stimulation using microfluidic devices is a promising method to regulate mechanical stress on intestinal organoids [17,18]. However, intestinal organoids are cystic structures with a single lumen that are more easily deformed than other types of spherical organoids, making them more susceptible to pressure in the microfluidic channel. Therefore, producing microfluidic trapping devices capable of immobilizing intestinal organoids under appropriate pressure would allow for the induction of intestinal organoid features, fusion, and surface topography in the microfluidic channel.

**Figure 1:**
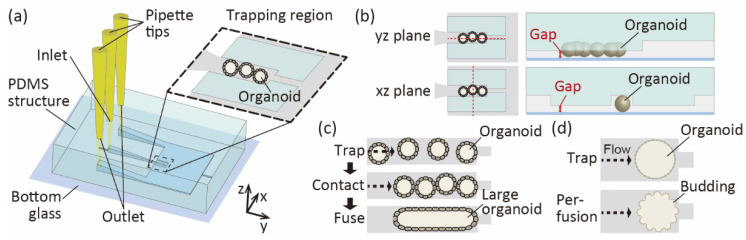
Conceptual illustration for manipulation of organoids using the proposed microfluidic device. (**a**) Microfluidic organoid-trapping device to trap and perfusion culture of organoids at its trapping region. (**b**) A top and side view of the trapping region. Red arrows indicate the wall gaps between the channel wall and the bottom surface. (**c**) Concept of organoid fusion in the trapping region. (**d**) Concept of applying fluidic stress to the organoid in the trapping region.

In this study, we propose a microfluidic organoid-trapping device to manipulate and stimulate human intestinal organoids (Figure 1a). The proposed device has a microchannel made of polydimethylsiloxane (PDMS) and bottom glass. When intestinal organoids are introduced into a microchannel, they are immobilized in the trapping region by clogging at the constriction point. The trapping region has gaps between the channel walls and bottom glass (Figure 1b). Since excess culture medium escapes from the gap, organoids are protected from high hydrostatic pressure. Using this device, we showed that human intestinal organoids can be trapped in a channel while controlling their number. To demonstrate the potential applications of the device for manipulating and stimulating organoids, we investigated whether their fusion could be induced to produce large organoids by alignment of organoids in the channel (Figure 1c). We induced the fusion of these organoids along channel dimensions since the fused organoids are known to exhibit similar properties to normal organoids [7,8]. Furthermore, we examined whether the device could apply fluidic shear stress to the organoids to regulate their surface structures by mechanical stress (Figure 1d).

## 2. Materials and Methods

### 2.1. Device Fabrication

The microfluidic organoid-trapping device was produced using a 3D-printed mold fabricated with a commercial stereolithography apparatus (Perfactory, EnvisionTec, Dearborn, MI, USA). The printed mold was exposed to UV light for complete curing using a laser machine (UV-LED, Keyence Corp., Osaka, Japan). After coating 2 µm parylene onto the mold with a chemical vapor deposition machine (Parylene Deposition System 2010, Specially Coating Systems Inc., Indianapolis, IN, USA), we placed the mold in a Petri dish and poured the PDMS elastomer (Sylgard 184 Silicone Elastomer, Dow Corning Toray Co. Ltd., Tokyo, Japan) mixed at a 10:1 base/crosslinker ratio with a mixer (conditioning mixer AR-100, THINKY). PDMS was solidified by heating at 60 °C for 2 h. We peeled off the hardened PDMS structure from the mold and created holes for the inlet and outlets using a biopsy punch. The PDMS structure was washed with ethanol and bonded to a cover glass (24 mm × 36 mm, Matsunami) through oxygen plasma treatment (plasma etching system model FA-1, SAMCO) for 5 s at 75 W. The PDMS structure bonded to the cover glass was heated at 70 °C for 90 min to tighten adhesion. It was sterilized using O_3_ gas and UV light (CoolCLAVE Plus Personal UV and Ozone Sterilizer, Gelantis). The device was coated with 2-methacryloyloxyethyl phosphorylcholine polymer (Lipidure, NOF Corporation, Tokyo, Japan) to block cell adhesion onto the channel surface. The device was soaked in phosphate-buffered saline (PBS) (Cell Science & Technology Institute Inc., Miyagi, Japan) and degassed overnight to remove air bubbles in the channel. The dimensions of these devices are listed in Figure S1.

### 2.2. Organoid Culture

The growth culture medium contained Advanced Dulbecco’s Modified Eagle’s Medium/F12 (DMEM/F12) (Thermo Fisher Scientific, Waltham, MA, USA) supplemented with 1% (*v*/*v*) penicillin/streptomycin (Thermo Fisher Scientific), 10 mM HEPES (Thermo Fisher Scientific), 2 mM GlutaMAX (Thermo Fisher Scientific), 1 × B-27 Supplement (Thermo Fisher Scientific), 10 nM gastrin I (Sigma-Aldrich, St. Louis, MO, USA), 1 mM N-acetylcysteine (Sigma-Aldrich), 100 ng/mL recombinant mouse Noggin (PeproTech, Cranbury, NJ, USA), 50 ng/mL recombinant mouse epidermal growth factor (Thermo Fisher Scientific), 100 ng/mL recombinant human Insulin-like Growth Factor-1 (BioLegend, San Diego, CA, USA), 50 ng/mL recombinant human fibroblast growth factor-basic (FGF- 2) (PeproTech), 1 μg/mL recombinant human R-spondin1 (R&D Systems, Minneapolis, MN, USA), 500 nM A83-01 (Tocris), and 20% (*v*/*v*) Afamin/Wnt3a conditioned medium (JSR Life Sciences, Sunnyvale, CA, USA).

Human intestinal epithelial cells (InEpc, cc-2931, Lonza) were embedded in Matrigel, as previously described [4]. They were suspended in Matrigel (growth factor-reduced, Corning) at 1000 cells/20 μL and dispensed into 48-well plates (tissue culture-treated, IWAKI) at 20 μL/well with a pipette. Matrigel was gelled in a 37 °C, 5% CO_2_ incubator for 10 min, and 300 μL of the growth medium was added to each well. Subsequently, the 48-well plates were cultured in an incubator. The growth medium was changed every other day. After a week of culturing, the spherical organoids were approximately 500 μm in diameter.

To subculture organoids, we aspirated the growth medium from the wells and dissolved the organoid-laden Matrigel using 500 μL/well TrypLE Express (Thermo Fisher Scientific). After 5–10 min of incubation at 37 °C, a suspension of human intestinal epithelial cells was obtained. The cells were washed with PBS and suspended in Matrigel at 1000 cells/20 μL. Matrigel-containing cells were dispensed into a 48-well plate at a density of 20 μL/well and incubated for 10 min. After gelation, growth medium containing 10 μM Y-27632 (FUJIFILM Wako Pure Chemical Corporation) was added to each well at 300 μL/well.

### 2.3. Organoid Fusion

All pipette tips, tubes, and cell strainers used in this study were coated with 10% fetal bovine serum (FBS) (Thermo Fisher Scientific) to prevent cell adhesion. Mature organoids were extracted from Matrigel using a cell recovery solution (Corning). The organoids–100–200 μm in diameter were selected using cell strainers (PluriStrainer). 

**Figure 2:**
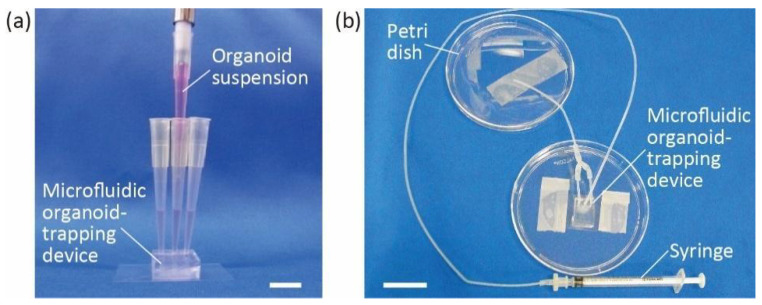
Experimental setup for organoid trapping and perfusion. (**a**) Introducing organoid suspension into the inlet of microfluidic organoid-trapping device. (**b**) Microfluidic organoid-trapping device connected to a syringe and tubes for organoid perfusion. Scale bars are (**a**) 1 cm and (**b**) 2 cm.

Three pipette tips were inserted into the inlet and outlet of the degassed PDMS microfluidic organoid-trapping device. DMEM/F12 containing 10 mM HEPES and 1% (*v/v*) penicillin/streptomycin was then gently poured into the inlet. The PBS was replaced in the microchannel, and the device was filled. PBS was pushed away to the outlets and sucked up with a pipette. Organoids of 100–200 μm were poured into the microchannel through the inlet (Figure 2a) and trapped in the microchannel. Growth culture medium supplemented with 3% Matrigel was added to the channel from the inlet, and excess medium was removed from the outlets. Organoids in the microchannel were incubated at 37 °C in a 5% CO_2_ atmosphere.

### 2.4. Perfusion Culture for Organoids

A single organoid, approximately 500 μm in diameter, was selected for better visibility and trapped in the microchannel in almost the same manner as described in the previous section. Briefly, we trapped an organoid in the microchannel, and the device was filled with growth culture medium containing 3% Matrigel. We removed the pipette tips from the inlet and outlets and inserted the end of the ethylene tetrafluoroethylene tube (inner diameter: 1.0 mm, outer diameter: 1.5 mm) connected to a 1 mL syringe (Terumo Japan) into the inlet. The drain tubes leading to a Petri dish were connected to the outlets. The syringe was pressurized using a pump (KDS200, KD Scientific, Inc., Holliston, MA, USA) to infuse the growth culture medium into the device at a rate of 10 μL/h for 70 h (Figure 2b). During perfusion, the organoid trapped in the device was incubated at 37 °C in a 5% CO_2_ atmosphere with the pump placed outside the incubator. Through the tube connecting the syringe to the device, culture medium was supplied from outside to inside the incubator and warmed up before reaching the device.

### 2.5. Staining

To label actin and cell nuclei, organoids were incubated with Alexa Fluor 488/647 phalloidin conjugate (Thermo Fisher Scientific) and Hoechst 33342 (Thermo Fisher Scientific), respectively. For immunostaining of lysozyme, organoids were fixed with 4% paraformaldehyde (Muto Pure Chemicals, Co.) for 30 min at room temperature. After washing with PBS, the organoids were permeabilized with 1% saponin (FUJIFILM Wako Pure Chemical Corporation) and 0.3% Triton X-100 (Alfa Aesar) for 20 min, and blocked with 1% (*v/v*) bovine serum albumin (Sigma-Aldrich) in PBS for 30 min. The organoids were then incubated with recombinant rabbit anti-Lysozyme antibody (ab108508; Abcam) at 4 °C overnight. After washing, the cells were incubated with Alexa Fluor 488 goat anti-rabbit IgG secondary antibody (Thermo Fisher Scientific) at room temperature for 2 h. To observe the stained organoids, we used an optical microscope (IX71, Olympus Life Sciences) and a confocal laser microscope (LSM780, Carl Zeiss).

### 2.6. Image Analysis

All image analyses were performed using ImageJ software (National Institutes of Health, Bethesda, MD, USA). In measuring the areas of trapped organoids, we manually set the outline of organoids in the channel and measured the cross-sectional area. To measure the number of buddings, we prepared Z-stack images of the organoids and manually counted the number of buddings using ImageJ software. During the measurement, we counted an uneven structure as budding when we confirmed that the angle of the actin arc was greater than 180°. This procedure was repeated more than twice to ensure the absence of manual errors.

### 2.7. Particle Image Velocimetry

To evaluate the flow velocities loaded on the organoids in the trapping region of the microfluidic device, we investigated the distributions of flow velocities in the channel using particle image velocimetry (PIV). Fluorescent beads 1 μm in diameter (Fluoresbrite YG Microspheres, Polusciences) were suspended in PBS. We infused bead-laden PBS at 10 μL/h into the microchannels and recorded the motion of the beads using an optical microscope. We analyzed the recorded video using particle-tracking software (Flownizer2D2C, Ditect) to calculate the flow velocity distributions.

### 2.8. Statistical Analysis

Multiple comparisons of the relative areas of the trapped organoids during culture were performed using one-way analysis of variance (ANOVA). Moreover, pairwise differences were evaluated using the Tukey–Kramer test. These statistical analyses were conducted using the statistical software R.

## 3. Results

### 3.1. Characteristics of the Microfluidic Organoid-Trapping Device

**Figure 3:**
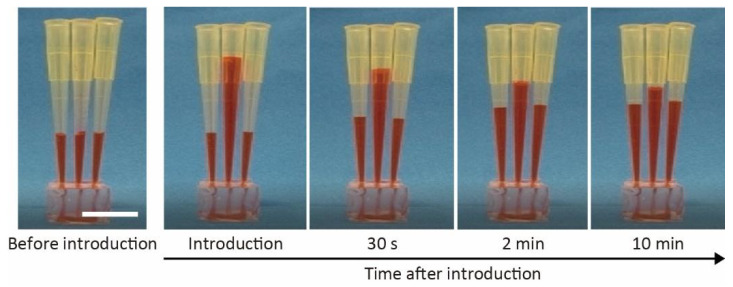
Time-lapse images of introducing water containing red ink into the microchannel from an inlet. Scale bar is 1 cm.

We monitored the introduction of colored water as an alternative to the culture medium (Figure 3) to verify the characteristics of the microfluidic organoid-trapping device. We poured 200 μL of colored water into the pipette tip inserted at the inlet, which flowed into the microchannel under hydrostatic pressure. The flow rate at the inlet decreased as the water moved from the inlet to the outlets, and eventually stopped within several hours. This result indicates that pipette tips allow for easy introduction of the culture medium and organoids into the microchannel without additional equipment, such as pumps and syringes.

**Figure 4:**
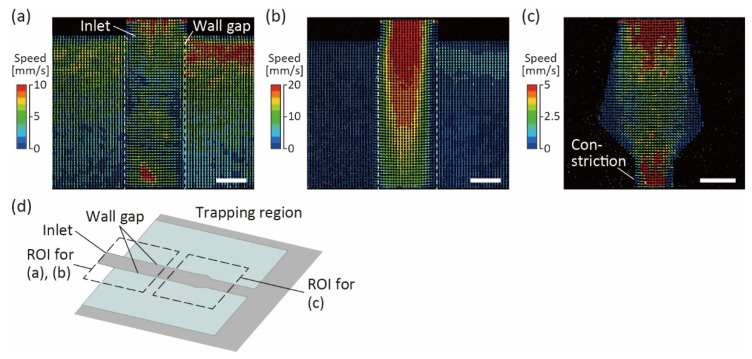
Bottom-view images with color vectors representing the speeds of fluorescent beads in the trapping region of the device. White dotted lines indicate the wall gaps in the channel. (**a**) Inlet of the trapping region near the bottom. (**b**) Inlet of the trapping region, at a height of more than 50 μm from the bottom. (**c**) Downstream of the trapping region near the bottom. (**d**) Schematic diagram for the trapping region, indicating the region of interest (ROI) for the vectors shown in (**a**–**c**). Scale bars are 100 μm.

To evaluate the flow characteristics in the trapping region of the channel, we perfused PBS with fluorescent beads at 10 μL/h and measured their flow velocities using PIV (Figure 4). At the trapping region near the bottom surface, flows in two directions were observed: the flow downstream along the trapping region, and the flow escaping from the trapping region through the wall gaps (Figure 4a). Only downstream flows were observed at heights above 50 μm from the bottom glass, because the PDMS wall prevented the flows from escaping the trapping region (Figure 4b). The results showed that the wall gaps enabled the excess medium to diffuse, preventing organoids from high pressure and deformation. Moreover, downstream of the trapping region, even near the bottom surface, we confirmed that almost no flow escaped from the trapping region, and most flows were directed downstream of the trapping regions (Figure 4c). This result indicates that when organoids are introduced into the trapping region, they can be trapped at a constriction downstream of that region.

### 3.2. Fusion of Intestine Organoids in the Microfluidic Device

**Figure 5:**
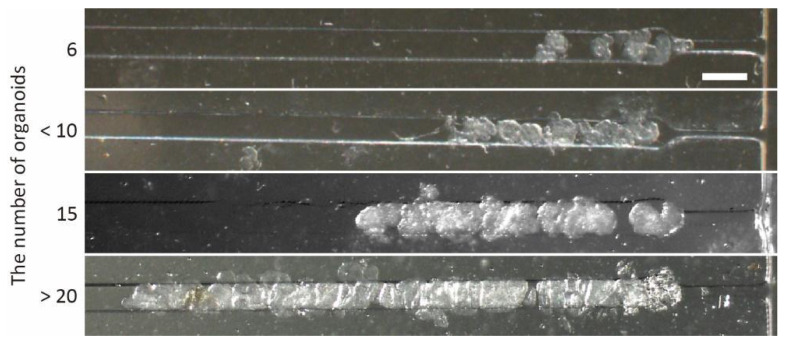
Trapped organoids at the trapping region of microfluidic devices. Scale bar is 200 μm.

To investigate organoid trapping using the microfluidic device, we prepared intestinal organoids 200 μm in diameter. After the organoid suspension was loaded into the inlet tips, the organoids were introduced into the trapping region using hydrostatic pressure. Finally, the organoids were immobilized in the trapping region. As a result, we confirmed that all organoids over 100 μm in diameter were trapped at the constriction of the trapping region in the order they entered the region (Figure 5), because the flows could reach the end of trapped organoids in the channel even when multiple organoids were trapped (Figure S2). These results indicate that the microfluidic device driven by hydrostatic pressure is an effective tool for trapping organoids in the microfluidic channel without additional apparatus, leading to easy control of the number of trapped organoids.

**Figure 6:**
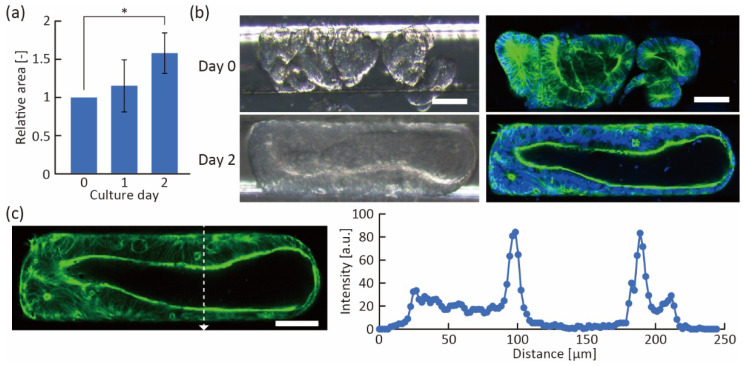
Fusion of trapped organoids. (**a**) The relative area of the trapped organoids calculated from microscope images (*n* = 4, mean ± s.d., * *p* < 0.05, Tukey–Kramer test). (**b**) Bright-field (left) and fluorescent (right) images of trapped organoids at 0 and 2 days of culture. Green shows actin and blue shows cell nuclei. (**c**) Profile of fluorescent intensity in the white dotted line of the fluorescent image (shown in Figure 6b), indicating actin. Scale bars are 100 μm.

Furthermore, we induced the fusion of the trapped organoids in the trapping region. In fusion induction, after trapping the organoids in a straight line, the growth medium supplemented with 3% Matrigel was used to fill the region because its viscosity was suitable for immobilizing the organoids even under medium exchange. The hydrostatic pressure and viscosity of the medium with 3% Matrigel allowed the organoids to remain in contact for several days. As a result, the trapped organoids continued to grow and their volume in the channel increased (Figure 6a), indicating that sufficient nutrition was supplied to the organoids. After two days of incubation, we found that multiple organoids had fused into a single large tube-like organoid (Figure 6b and Figure S3). The fused tube-like organoid had a thick actin layer, and actin was highly expressed on the luminal side of the organoids (Figure 6c), indicating that the fused organoid had maintained its proper polarity. In addition, we were able to obtain large organoids in a shorter time (2 days) than the conventional method (7 days) [4], suggesting that we can obtain fused organoids with less cellular debris accumulated in the organoid as the culture progressed. Because the debris interferes with the uniform diffusion of chemicals, the tube-like fused organoids constructed with our device have the potential to be useful tools for drug screening and biological analysis.

### 3.3. Morphological Changes in Intestinal Organoids Cause by Flow Stimulation

**Figure 7:**
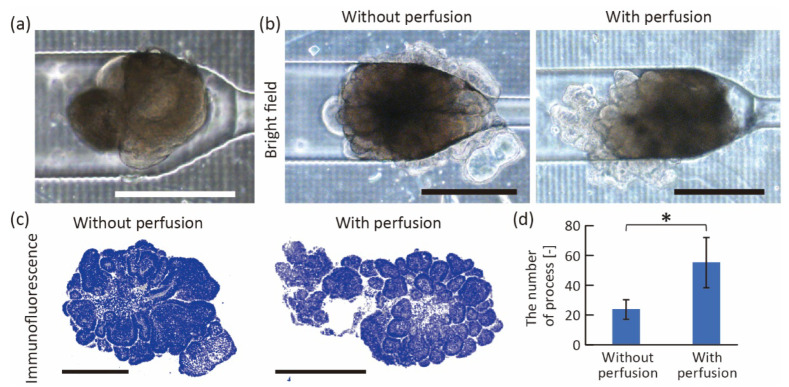
Perfusion culture of trapped organoids. (**a**) Image of trapped organoid before perfusion. (**b**) Bright-field and (**c**) fluorescent images of the organoids after 70 h culture in the device with and without perfusion. White shows actin and blue shows cell nuclei. (**d**) Comparison of the number of buddings in the intestinal organoids with and without the perfusion culture (*n* = 4, mean ± s.d., Students’ *t*-test, *p* < 0.05). Scale bars are 500 μm.

To demonstrate the stimulation of intestinal organoids using the proposed device, we perfused a growth medium to apply fluidic stress to a trapped organoid. After introducing a single organoid into the device by hydrostatic pressure (Figure 7a), we infused the growth culture medium containing 3% Matrigel at 10 μL/h using a syringe pump. On the other hand, the organoids cultured without perfusion were prepared in the device by exchanging the medium with 3% Matrigel every other day. After 70 h of culturing, we confirmed that both organoids grew in the trapping region, and some cells migrated into the wall gaps (Figure 7b). Interestingly, the surface of the perfused organoid became rougher, and the number of buddings increased (Figure 7c,d). In the conventional study, when seeding intestinal organoids on the 2D substrate and perfusing culture medium on the basal side of organoids, villus-like structures were constructed on their surfaces [19]. Therefore, the results indicated that perfusion of the organoids showed the same increase in budding as when they were perfused in a two-dimensional plane.

**Figure 8:**
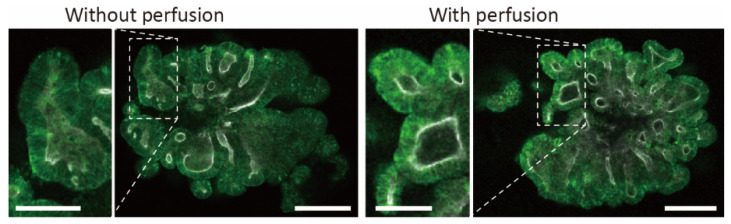
Budding formation of perfused organoids, showing localization of lysozyme (paneth cell marker) in the intestinal organoid with and without perfusion culture. Green shows lysozyme and white shows actin. Scale bars are 200 μm (overall image) and 100 μm (magnified image).

To characterize the budding, we visualized lysozyme (a Paneth cell marker) by immunostaining. As shown in Figure 8, lysozyme was observed at the tip of the increased budding. As lysozyme is known to localize at crypts [20], these results indicate that the acceleration of crypt-like domain formation caused morphological changes. Conventional research has shown that villus-like domains increase when fluidic stress is applied to the basal side of intestinal organoids cultured on 2D porous membranes [19]. Although the factors causing the formation of crypt-like domains instead of villus-like domains are unclear, continuous medium supply or fluidic shear stress under perfusion could be influential in the proposed microfluidic devices. Thus, the mechanism of crypt-like domain formation remains a topic for future work. However, perfusion culture using the proposed device can be used in biological studies to promote budding formation in intestinal organoids. 

## 4. Conclusions

We propose a simple microfluidic device to trap, fuse, and stimulate human epithelial cell-derived intestinal organoids. The organoids were trapped and aligned in the device under hydrostatic pressure without any obvious deformation. We demonstrated that the trapped organoids were fused in the device within two days and formed large tube-like organoids. Furthermore, applying flow to the basal side of the organoid increased the number of buddings. Thus, we believe that our device will be a useful tool for various investigations of intestinal organoids, such as studying the effects of chemical and flow stimulation on single and tube-like organoids. There is still room for improvement in taking out intestinal organoids from microfluidic devices. To extract the organoids, the device must be separated from the bottom glass, or the organoids must be sucked out of the inlet, both of which are difficult to maintain sterile conditions and avoid deformation of the organoids. Therefore, we believe that the construction of a microfluidic system with openable ceilings for collecting the intestinal organoids will be a future challenge for further investigation. The proposed device could also be applied to other spherical and cystic organoids. Therefore, biological and biomedical researchers will widely use microfluidic organoid-trapping devices as standard culture systems for organoids.

## Data Availability

The authors declare that all data supporting the findings of this study are available in the paper.

## Supplementary Materials

The following supporting information can be downloaded at: https://www.mdpi.com/article/10.3390/mi13122082/s1, Figure S1: Dimensions of a microfluidic channel in the organoid-trapping device; Figure S2: Flow simulation in the trapping region of proposed microfluidic organoid-trapping device; Figure S3: Fluorescent orthoimage of a tube-like organoid at 2 days of culture.
