# Supplementary material for: Microfluidic Device to Manipulate 3D Human Epithelial Cell-Derived Intestinal Organoids
Source: Micromachines (Basel). 2022 Nov 26;13(12):2082. doi: 10.3390/mi13122082 (PMC9785580; doi:10.3390/mi13122082)
Supplement: Supplementary file 1 [file micromachines-13-02082-s001.zip › micromachines-1973637-supplementary.pdf]

micromachines

*Supporting Information*

# Microfluidic Device to Manipulate 3D Human Epithelial cell-derived Intestinal Organoids

**Miki Matsumoto, Yuya Morimoto, Toshiro Sato and Shoji Takeuchi**

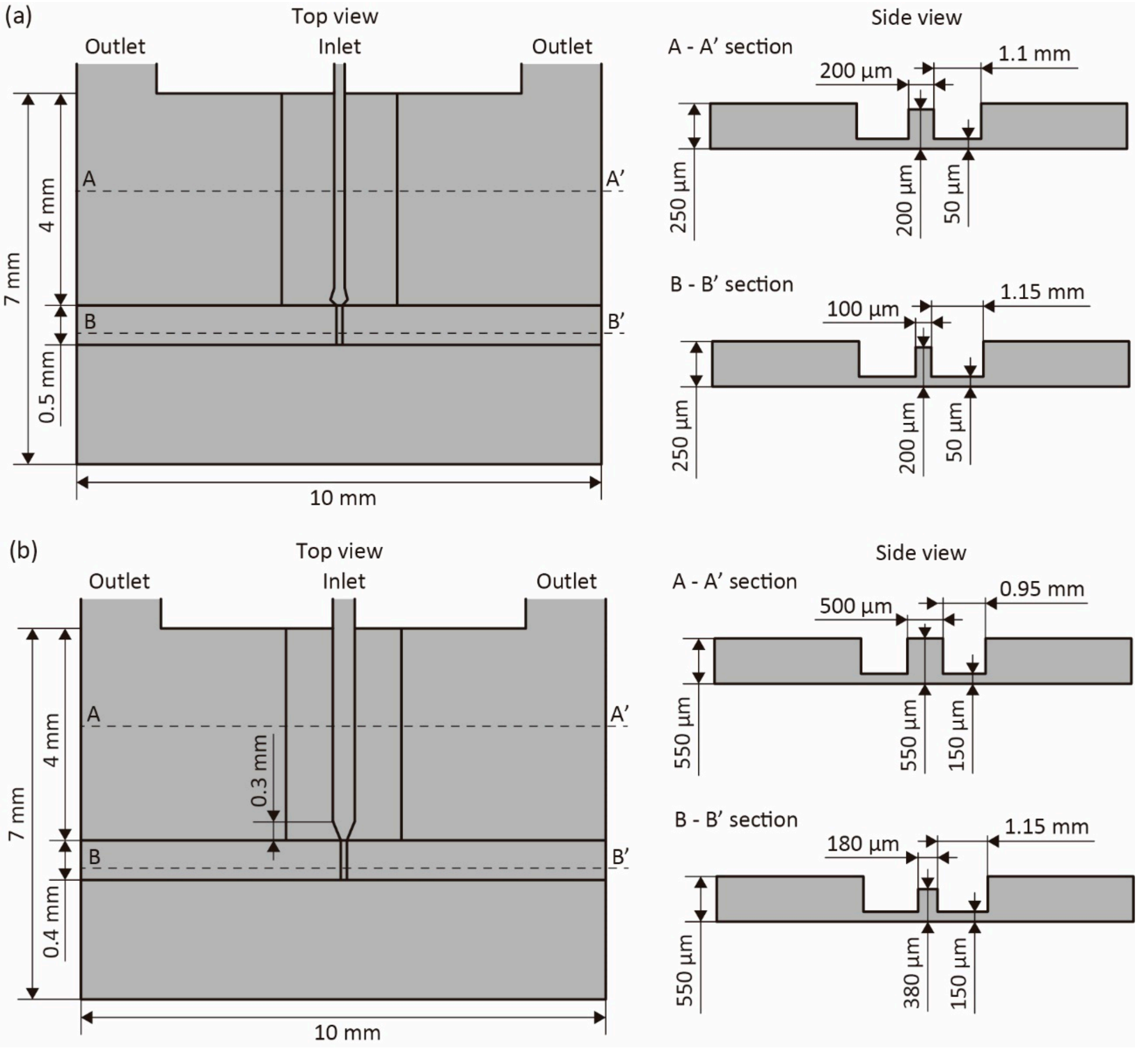


**Scheme 1.** Dimensions of a microfluidic channel in the organoid-trapping device for (a) organoid fusion and (b) perfusion culture.

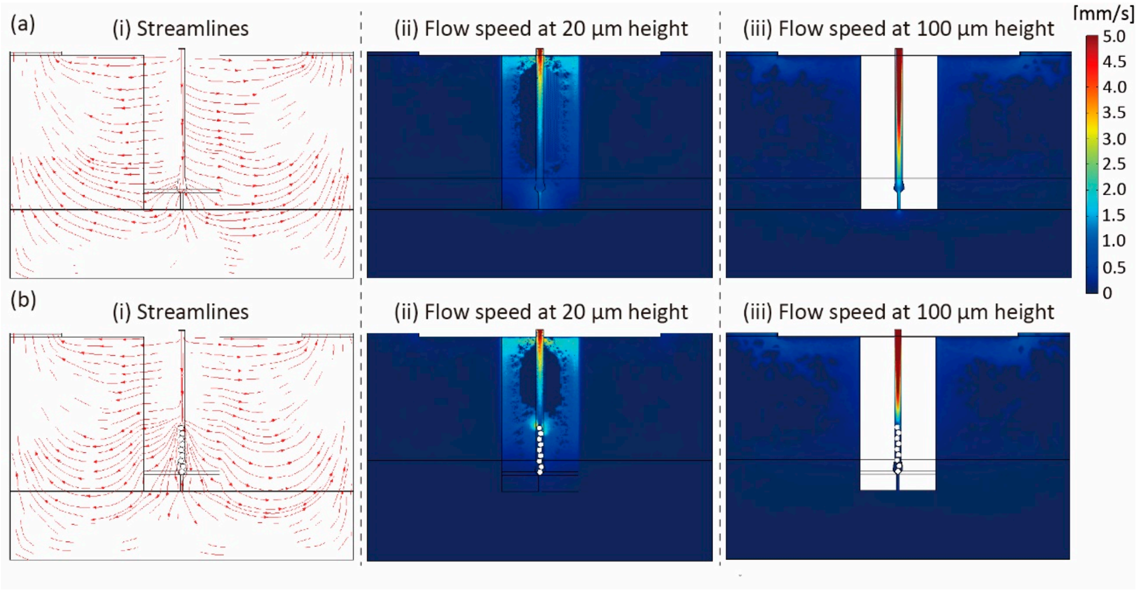


**Scheme 2.** Flow simulation in the trapping region of proposed microfluidic organoid-trapping device (a) when no organoid was in the trapping region and (b) when organoids were clogged. (i) steamlines shown with red lines with arrows, and colormap for flow speed at (ii) 20 µm height and (iii) 100 µm height in the channel.

For the flow simulation, we prepared 3D static models of the microfluidic channel using the finite element method (FEM) simulation software (COMSOL Multiphysics, COMSOL Inc.). The flow simulation was performed under the condition that the inlet flow speed was 8.4 mm/s and the organoids represented as immovable obstacles adhered to the channel walls.

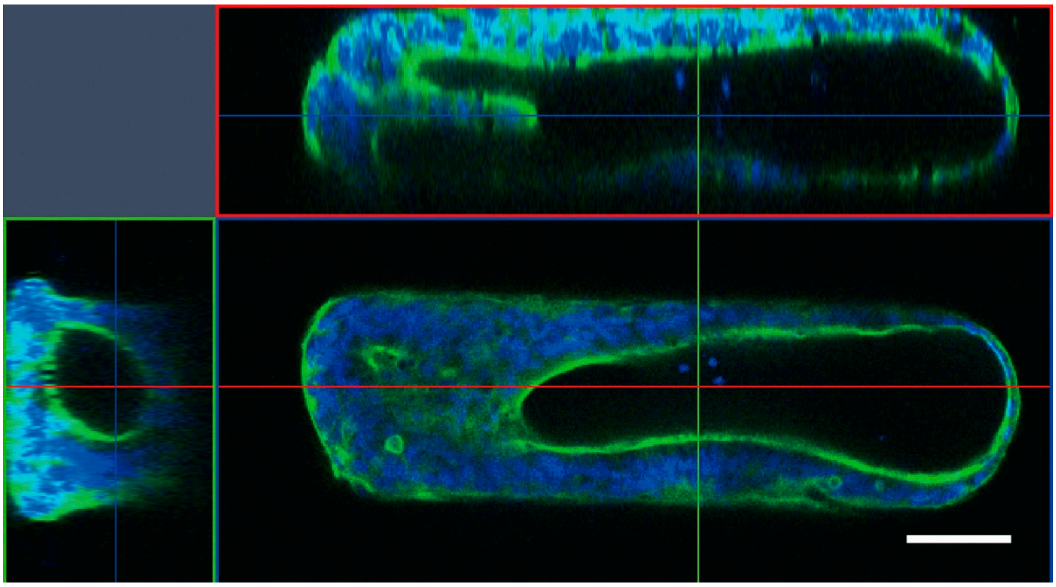

**Scheme 3.** Fluorescent orthoimage of a tube-like organoid at 2 days of culture. Green shows actin and blue shows cell nuclei. Scale bars is 100 µm.
